# Functional reorganization in obstructive sleep apnoea and insomnia: A systematic review of the resting-state fMRI

**DOI:** 10.1016/j.neubiorev.2017.03.013

**Published:** 2017-06

**Authors:** Habibolah Khazaie, Mattia Veronese, Khadijeh Noori, Farnoosh Emamian, Mojtaba Zarei, Keyoumars Ashkan, Guy D. Leschziner, Claudia R. Eickhoff, Simon B. Eickhoff, Mary J. Morrell, Ricardo S. Osorio, Kai Spiegelhalder, Masoud Tahmasian, Ivana Rosenzweig

**Affiliations:** a Sleep Disorders Research Center, Kermanshah University of Medical Sciences (KUMS), Kermanshah, Iran; b Sleep and Brain Plasticity Centre, Department of Neuroimaging, IoPPN, King’s College, London, UK; c Department of Psychiatry, University of Social Welfare and Rehabilitation Sciences, Tehran, Iran; d Institute of Medical Sciences and Technology, Shahid Beheshti University, Tehran, Iran; e School of Cognitive Sciences, Institute for Research in Fundamental Sciences (IPM), Tehran, Iran; f Department of Neurosurgery, King’s College Hospital, London, UK; g Sleep Disorders Centre, Guy’s and St Thomas’ Hospital, London, UK; h Institute of Neuroscience and Medicine (INM-1), Research Center Jülich, Jülich, Germany; i Department of Psychiatry, Psychotherapy, and Psychosomatics, RWTH Aachen University, Aachen, Germany; j Institute of Clinical Neuroscience & Medical Psychology, Heinrich Heine University Düsseldorf, Düsseldorf, Germany; k Academic Unit of Sleep and Breathing, National Heart and Lung Institute, Imperial College London, UK and NIHR Respiratory Disease Biomedical Research Unit at the Royal Brompton and Harefield NHS Foundation Trust,Sydney Street, London, SW3 6NP, UK; l Center for Brain Health, NYU School of Medicine, New York, NY, United States; m Department of Clinical Psychology and Psychophysiology/Sleep Medicine, Center for Mental Disorders, University of Freiburg Medical Center, Freiburg, Germany

**Keywords:** Resting-state fMRI, Obstructive sleep apnea, Insomnia disorder, Sleep disorders, Major depressive disorder

## Abstract

Functional neuroimaging techniques have accelerated progress in the study of sleep disorders. Considering the striking prevalence of these disorders in the general population, however, as well as their strong bidirectional relationship with major neuropsychiatric disorders, including major depressive disorder, their numbers are still surprisingly low. This review examines the contribution of resting state functional MRI to current understanding of two major sleep disorders, insomnia disorder and obstructive sleep apnoea. An attempt is made to learn from parallels of previous resting state functional neuroimaging findings in major depressive disorder. Moreover, shared connectivity biomarkers are suggested for each of the sleep disorders. Taken together, despite some inconsistencies, the synthesis of findings to date highlights the importance of the salience network in hyperarousal and affective symptoms in insomnia. Conversely, dysfunctional connectivity of the posterior default mode network appears to underlie cognitive and depressive symptoms of obstructive sleep apnoea.

## Highlights

• Resting state functional MRI studies is a promising non-invasive tool for better understanding of the pathophysiology of sleep disorders.
• The salience network is involved in hyperarousal and affective symptoms in insomnia.
• The posterior default mode network appears to underlie cognitive and depressive symptoms of obstructive sleep apnoea.
• Disruption of intrinsic networks have been demonstrated in major depression, which is a common co-morbidity of sleep disorders.

## 1 Introduction

It is known that sleep serves a restorative function for the brain (Mander et al., 2016), emotions and cognition (Goldstein and Walker, 2014), and that it involves dramatic changes to our perception and consciousness (Deco et al., 2014). Even a transient perturbation during sleep can have a lasting impact on intrinsic activity and responsivity during wake periods (Buzsaki and Watson, 2012). The prevalence of sleep-wake cycle disturbances in psychiatric and neurological diseases, such as major depressive disorder (MDD), is widely recognized (Morin et al., 2015b; Rosenzweig et al., 2017), and a possible role for sleep modulation as a therapeutic tool in several debilitating brain disorders has been reported (Landsness et al., 2011, Lustenberger et al., 2016, Mander et al., 2016). Today, the worldwide prevalence of the two most common sleep disorders, namely insomnia disorder and obstructive sleep apnea (OSA), is thought to be on the rise due to an aging population, and due to ever increasing demands and stressors of the “24/7” rhythm of the modern world (Lévy et al., 2015; Morin et al., 2015b). The hidden economical costs of those two sleep disorders to society, patients and their families, as well as impact on patients’ quality of life, increased propensity to workplace and traffic accidents, and increase in number of co-morbidities, have all been increasingly recognized (Morin et al., 2015, Rosenzweig et al., 2015).

Over the last decade, different functional neuroimaging techniques (fMRI), including resting-state fMRI (rs-fMRI), have been widely applied in sleep disorders to enhance the understanding of the pathophysiology and potential compensatory mechanisms at play (also see reviews (Desseilles et al., 2008, Spiegelhalder et al., 2015, Tahmasian et al., 2015a, Tahmasian et al., 2016a)). This review examines the contribution of rs-fMRI to our current understanding of their dysfunctional circuitry and explores neurocognitive similarities with synthesis of the blueprint findings in studies of their shared co-morbid psychiatric disorder, MDD (Baglioni et al., 2011, Gupta and Simpson, 2015).

## 2 Data source and study selection

**Fig 1:**
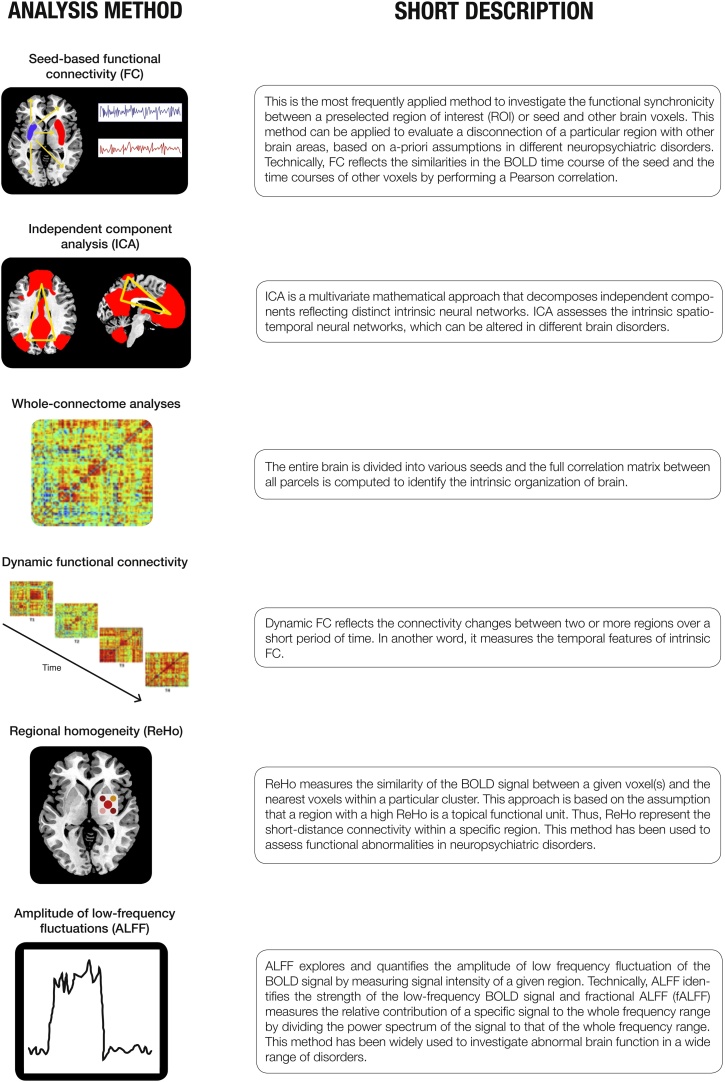
Short description of the most important analyses method for investigation of resting state functional MRI (rs-fMRI) findings (adapted from Tahmasian et al. (2015a)).

**Fig. 2:**
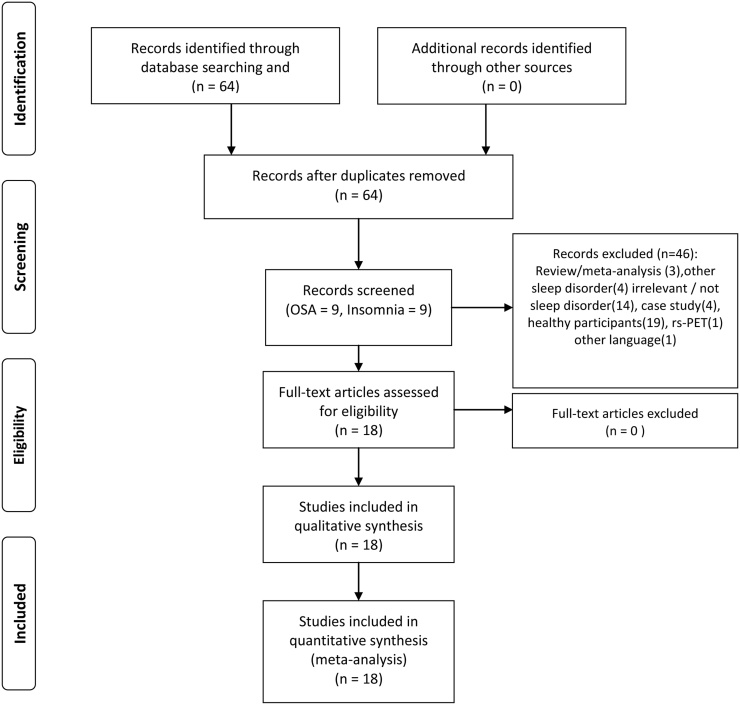
Paper selection strategy flow chart.

New network-based techniques allow us to identify large-scale brain networks aberrations in a variety of disorders by looking at changes in blood-oxygen-level-dependent (BOLD) signal using fMRI (Mulders et al., 2015, Tagliazucchi and Laufs, 2014). An important methodological development was their consistent identification during the “resting-state” condition, i.e. when a subject is not engaged in any external task (Biswal et al., 1995, Snyder and Raichle, 2012). This independence of task-based paradigms in rs-fMRI offers the important advantage of studying the intrinsic functional organization of the brain and is reproducible across different populations and study settings (Tagliazucchi and Laufs, 2014). Short descriptions of different approaches to rs-fMRI analysis that are referred to in this review are presented in Fig. 1. In this review, several recent studies that evaluated functional disturbances in sleep disorders using different rs-fMRI analysis methods are summarised, and their putative role in investigations is described further (Table 1). Based on the Preferred Reporting Items for Systematic Reviews and Meta-Analyses (PRISMA) statement (Moher et al., 2009), we conducted our search in the PubMed database in April 2016 to systematically explore studies using rs-fMRI in patients suffering from the two most prevalent sleep disorders, insomnia and OSA. Key words were “(resting-state functional magnetic resonance imaging OR resting-state fMRI) AND (sleep disorders OR sleep-related breathing disorders OR sleep apnea OR OSA OR insomnia)”. We screened the original English literature that was retrieved by the search string. A total number of 64 studies emerged from the literature search. Subsequently; we excluded studies that included only healthy controls; reviews; case reports; letters-to-editors; and studies that were not related to the main topic of the present review. Finally; we included 18 studies (9 for OSA; 9 for insomnia). Abnormalities of functional networks of the two sleep disorders were inferred from the findings of the independent component analysis (ICA) and seed-based functional connectivity (FC) analysis studies. Conversely; regional abnormalities were demonstrated from the regional homogeneity (ReHo) and amplitude of low-frequency fluctuation (ALFF) analyses of rs-fMRI of the reported studies. Based on the rs-fMRI approach; 8 studies applied seed-based FC; 3 conducted an ICA; 3 used ALFF; and 4 performed ReHo (Table 1; Fig. 2).

**Table 1:** Studies entered into the meta-analysis are listed based on the year of publication and further alphabetically for each year. BMI = Body Mass Index; FC = Functional connectivity; fMRI = Functional magnetic resonance imaging; OSA = Obstructive sleep apnea; rs-fMRI = Resting-state functional magnetic resonance imaging; VBM = Voxel-based morphometry.

	Author, year	Number of subjects (patients/controls)	Number of male subjects (patients/controls)	Age of patients/controls (Mean ± SD)	Type of disorder	Imaging modality	Covariates	global signal regression	motion correction method
1	Li et al., 2016a, Li et al., 2016b	40/40	40/40	38.6 ± 8.1/ 39.3 ± 7.5	OSA	seed-based FC	BMI and age	+	six head motion parameters, all participants showed a maximum displacement of <1.5 mm) and a maximum rotation of <1.5°.
2	Park et al. (2016a)	69/82	52/58	48.3 ± 9.2/ 47.6± 9.1	OSA	whole-brain FC	age and gender	+/−	six head motion parameters, adding the first derivatives of the motion parameters as covariates to minimize signal changes due to motion.
3	Park et al. (2016b)	67/75	51/56	48.0 ± 9.2/ 47.1 ± 9.3	OSA	seed-based FC	age and gender	+	six head motion parameters.
4	Li et al. (2014)	25/25	25/25	39.4 ± 1.7/ 39.5 ± 1.6	OSA	ALFF	age and years of education	–	six head motion parameters, all subjects with >1.5 mm maximum displacement and maximum rotation >1.5°were excluded.
5	Zhang et al. (2015)	24/21	24/21	44.6 ± 7.4/ 40.6 ± 11.4	OSA	seed-based FC	Age, framewise displacemen,BMI	+	six head motion parameters, all participants had a maximum displacement of <2 mm and maximum rotation of <2°.
6	Taylor et al. (2016)	19/17	15/11	58 ± 4/ 57 ± 4	OSA	ICA	mean frame-wise displacement	N/S	Standard motion correction using FMRIB’s Linear Imaging Registration Tool (FLIRT), dual-regression of mean frame-wise displacement.
7	Peng et al. (2014)	25/25	25/25	39.4 ± 1.7/ 39.5 ± 1.6	OSA	ReHo	Age	N/S	six head motion parameters, participants with >1.5 mm maximum displacement and maximum rotation >1.5° were excluded.
8	Santarnecchi et al. (2013)	19/19	16/14	43.2 ± 8/ 41 ± 6	OSA	ReHo	age, gender, total brain volume, and BMI	N/S	N/S
9	Zhang et al. (2013)	24/21	24/21	44.6 ± 7.4/ 40.6 ± 11.4	OSA	ICA, VBM	age	N/S	six head motion parameters, all participants had a maximum displacement of <2 mm and maximum rotation of < 2°.
10	Li et al., 2016a, Li et al., 2016b	55/44	24/11	39.18 ± 10.34/39.91 ± 9.43	insomnia	ALFF	Sex, age, education level		head motion <1.5 mm or 1.5° were included.
11	Regen et al. (2016)	20/20	8/8	42.7 ± 13.4/ 44.1 ± 10.6	insomnia	Seed-based FC	age, gender, Beck Depression Inventory and State-Trait Anxiety Inventory	–	six head motion parameters, additional movement correction was performed by censoring images with a framewise displacement sum of more than 0.5 mm.
12	Nie et al. (2015)	42/42	15/18	49.24 ± 12.26/ 49.14 ±10.2	Insomnia	Seed-based FC	age, gender, and education	–	six head motion parameters, subjects with >1.5 mm maximum displacement and maximum rotation >1.5° were excluded.
13	Dai et al. (2014)	42/42	15/18	49.21 ± 10.96/ 49.14 ± 10.2	Insomnia	ALFF	age, gender, and years of education	–	six head motion parameters, subjects with >1.5 mm maximum displacement and maximum rotation >1.5° were excluded.
14	Wang et al. (2015)	59/47	21/14	39.3 ± 10.7/ 40.0 ± 9.1	Insomnia	ReHo	head motions	N/S	six head motion parameters, none of subjects had maximum displacement of >1.5 mm and rotation of >°1.5.
15	Dai et al. (2014)	24/24	7/12	54.8 ± 9.8/ 52.5 ± 6.6	Insomnia	ReHo	age, gender, years of education	–	six head motion parameters, subjects with >1.5 mm maximum displacement and maximum rotation >1.5° were excluded.
16	Li et al. (2014)	15/15	7/7	41.3 ± 8.9/ 39.8 ± 11.2	Insomnia	Seed-based FC	–	N/S	six head motion parameters, none of the subjects had >1.5 mm maximum displacement and maximum rotation >1.5.°
17	Chen et al. (2014)	17/17	0/0	27.16/ 27.56	Insomnia	ICA	–	–	six head motion parameters, motion files were used to censor TRs in which the derivative value of any of 6 motion parameters exceeded Euclidean norm of 1.2.
18	Huang et al. (2012)	10/10	5/5	37.5/ 35.5	Insomnia	Seed-based FC	–	+	six head motion parameters, none of subjects had >1.5 mm maximum displacement or >1.5°.

## 3 Applications of rs-fMRI in OSA

OSA arises from recurrent partial or complete pharyngeal secession during sleep (Jordan et al., 2014, Lévy et al., 2015), and it may lead to cognitive decline, deficits in attention, working and episodic memory, executive functioning, and quality of life (Rosenzweig et al., 2017, Rosenzweig et al., 2016). Patients with OSA are also two to 13 times more likely to experience traffic accidents (Rosenzweig et al., 2017). It is considered as one of the rare modifiable risk factors for dementia(Osorio et al., 2015, Rosenzweig et al., 2015, Yaffe et al., 2014), with some studies also suggesting that its prevalence is higher in people with Alzheimer’s dementia (Emamian et al., 2016, Rosenzweig et al., 2017). Moreover, patients with OSA are reported to have higher rates of excessive daytime somnolence, lower work and school efficiency, dysfunctional interpersonal relationships, and a higher rate of work accidents (Rosenzweig et al., 2017). In addition, high comorbidity of OSA with several psychiatric disorders such as major depressive disorder, anxiety, and posttraumatic stress disorder has been reported (Gupta and Simpson, 2015, Sharafkhaneh et al., 2005).

**Fig. 3:**
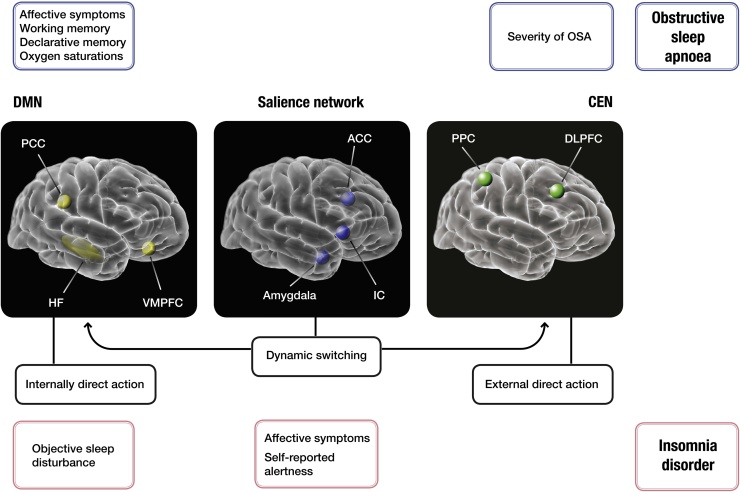
The schematic presentation of symptoms observed in patients with obstructive sleep apnoea (OSA) and insomnia disorder, and their correlations with the aberrant functional connectivity in hubs of three major intrinsic networks, as suggested by resting state functional MRI (rs-fMRI). Three major intrinsic brain networks are shown (from right to left): The aberrant connectivity of the frontoparietal network (including central executive network (CEN); green) has been linked to severity of OSA. It has key nodes in the dorsolateral prefrontal cortex (DLPFC) and the posterior parietal cortex (PPC). Its major task is in attentional selection of the relevant stimuli, and any disturbance in this network is likely to have a domino effect on other three major intrinsic networks. Its malfunctioning could lead to executive deficits in some patients with OSA. On the other hand, salience signals are integrated in the salience network (blue), which has been affected in both OSA and insomnia. This network has a central role in the detection of behaviorally relevant stimuli and the coordination of neural resources. It includes the insular cortices (IC) and the anterior cingulate cortex (ACC). In OSA, links between this network and increased sympathetic outflow have been reported. Similarly, in insomnia, correlation with hyperarousal and affective symptoms has been suggested. The salience network via IC mediates the ‘switching' between activation of the CEN and final major intrinsic the default-mode network (DMN; yellow) to guide appropriate responses to salient stimuli (adapted from Uddin (2015)). The DMN has key nodes in the posterior cingulate cortex (PCC) and the ventromedial prefrontal cortex (VMPFC). The DMN and CEN support self-related (or internally directed) and goal-oriented (or externally directed) cognition, respectively. In OSA, link between affective and cognitive symptoms (e.g. deficits in working and declarative memory) and aberrant connectivity of the posterior DMN has been shown. In insomnia, in contrast, the connectivity within this network is more closely correlated with objective sleep disturbance parameters. (For interpretation of the references to color in this figure legend, the reader is referred to the web version of this article.).

Recent rs-fMRI studies in OSA have demonstrated that long-term exposure to oxidative stress, intermittent hypoxia, hypo- and hypercapnia and sleep fragmentation, some of the major culprits behind OSA brain injury, may lead to significant global and regional connectivity deficits, especially in the default-mode network (DMN) and regions involved in the arousal and sensorimotor systems (Fig. 3). Taken together, rs-fMRI studies to date suggest several dysfunctional networks as a possible fingerprint of OSA (Fig. 3).

### 3.1 Abnormalities of functional networks in OSA

Alterations of intrinsic neural networks in patients with OSA have been investigated in six studies listed in Table 1 (Li et al., 2016b, Park et al., 2016a, Park et al., 2016b, Taylor et al., 2016, Zhang et al., 2015, Zhang et al., 2013). In the study by Zhang et al. (2013), seven networks of interest were identified and it has been reported that patients with OSA showed significant reduced FC within the anterior DMN, bilateral fronto-parietal network, sensorimotor network and increased FC within the posterior DMN compared to healthy controls (Zhang et al., 2013). A smaller grey matter volume in the medial prefrontal cortex (PFC) and left dorsolateral PFC was also reported (Zhang et al., 2013). Moreover, they were able to show a significant association between the altered FC of the right fronto-parietal network and the severity of OSA (Zhang et al., 2013). In another study, FC between each pair of DMNs’ sub-regions was explored in more details (Li et al., 2016b). Here, lower connectivity between the right hippocampus and the posterior cingulate cortex (PCC), medial PFC, and left medial temporal lobe (MTL) was shown (Li et al., 2016b). Of note, FC disruption between these areas has been in the past associated with cognitive impairment (Klupp et al., 2015, Pasquini et al., 2015, Tahmasian et al., 2015c). In addition, Li et al., 2016a, Li et al., 2016b demonstrated a positive link between FC of the right hippocampus-left MTL and rapid eye movement (REM) sleep and a negative link between FC of the PCC-right hippocampus and delayed memory in OSA (Li et al., 2016b).

Park et al., 2016a, Park et al., 2016b have assessed voxel-wise FC of the insular cortices in drug-naïve OSA patients (Park et al., 2016b). Here, FC disruption between the insula and many other regions including frontal, parietal, temporal, cingulate, limbic, basal ganglia, thalamus, occipital, cerebellar, and brainstem regions was found (Park et al., 2016b). Such findings are consistent with previous literature of other imaging modalities (Tahmasian et al., 2016a, Weng et al., 2014). For example, an ALE meta-analysis on voxel-based morphometry studies revealed significant atrophy in the bilateral parahippocampal and frontotemporal regions in patients with OSA (Weng et al., 2014). Using a similar approach in assessing convergent findings of structural and functional studies of OSA, our group demonstrated deficits in functioning and hypotrophy in the right basolateral amygdala and hippocampus, and the right central insula in patients with OSA (Tahmasian et al., 2016a). It has been further argued that FC alterations of insular cortices may singularly, or in synchrony with other CNS deficits, give rise to numerous autonomic, affective, sensorimotor, and cognitive dysfunctions that have been reported in patients with OSA (Tahmasian et al., 2016a). Accordingly, FC disruptions of insular cortices have been reported correlated to several sleep-related, cognitive and psychological parameters (Park et al., 2016b).

The insular cortex (Fig. 3) is a main node of the salience network (SN) that has a critical role in the detection and screening of emotional stimuli (Uddin, 2015) and its localized atrophy has been reported in OSA previously (Joo et al., 2013). The anterior insula in particular plays an important role in mediating dynamic interactions between large-scale brain networks involved in internal-oriented tasks (i.e. DMN) and external-oriented tasks (i.e. central executive network (CEN)) (Fig. 3) (Menon and Uddin, 2010). In keeping, Zhang et al. (2015) performed a seed-based FC analysis between the anterior insula as a seed, and the main hubs of the DMN and the CEN (Zhang et al., 2015). The findings suggested that in OSA patients, FC between the right insula and the DMN is disrupted and that this abnormal FC is associated with the severity of OSA (Zhang et al., 2015). In addition, the functional disconnection between the right insula and the PCC, as a main hub of the DMN, was linked with depressive scores and poorer working memory performance of OSA patients (Zhang et al., 2015). The group comparison pointed to no significant FC disruption between the right insula and the CEN (Zhang et al., 2015).

In view of these findings disruption of FC in the DMN may represent an important potential biomarker of the rs-fMRI findings in OSA. This also has biological plausibility, given that the DMN presents an important network for episodic memory and overall cognitive functioning (Greicius et al., 2003, McCormick et al., 2014), both of which have been also frequently reported as variably impaired in patients with OSA (Osorio et al., 2015, Rosenzweig et al., 2017).

Topological characteristics of functional brain networks have also been investigated using graph analysis (Park et al., 2016a). For example, Park et al., 2016a, Park et al., 2016b demonstrated abnormal FC in patients with OSA in several regions, including the cerebellar, frontal, parietal, temporal, occipital, limbic, and basal ganglia (Park et al., 2016a). In particular, aberrant cerebro-cerebellar FC was observed (Park et al., 2016a). Patients with OSA compared to heathy subjects, showed overall less efficient integration across the whole-brain areas and reduced regional topological characteristics of functional integration and specialization characteristics in regions showing disrupted FC (Park et al., 2016a). In addition, the link between the SN and sympathetic outflow in OSA has been explored by using ICA and resting post-ganglionic muscle sympathetic nerve activity (MSNA) (Taylor et al., 2016). A specific positive correlation between burst incidence and FC of the SN was reported (Taylor et al., 2016). This finding was not linked to the severity of OSA and the sleep state of OSA patients. Similarly, there was no association between burst incidence and FC of the DMN or sensorimotor networks (Taylor et al., 2016).

### 3.2 Regional abnormalities in OSA

Three studies demonstrated local functional differences in patients with OSA compared to healthy subjects (Dai et al., 2015, Peng et al., 2014, Santarnecchi et al., 2013). Using ReHo analyses, a significant lower coherence in the right temporal, parietal and frontal areas and significant higher coherence in the bilateral thalamic,somatosensory and motor regions has been observed in patients with severe OSA (Santarnecchi et al., 2013). These findings suggested a homeostatic reorganization of brain regions due to OSA with possible adaptive and maladaptive functional outcomes (Santarnecchi et al., 2013). Another OSA study, found a decrease of ReHo in the main hubs of DMN (Peng et al., 2014). However, higher ReHo was reported in the right posterior lobe of the cerebellum, right cingulate gyrus, and bilateral cluster covering the lentiform nucleus, putamen, and insula. The lower mean ReHo value in the right cluster of the precuneus and angular gyrus had a negative correlation with sleep time, and higher ReHo in the right posterior lobe of the cerebellum showed a positive link with slow wave sleep and in the right cingulate gyrus showed a positive correlation with the REM sleep (Peng et al., 2014). Overall, patients with OSA showed significant regional spontaneous activity deficits in the DMN sub-regions, leading authors to suggest that ReHo method might prove an useful noninvasive imaging tool for detection of early changes in cerebral ReHo in patients with OSA (Peng et al., 2014). Similarly, Li et al., 2016a, Li et al., 2016b reported deficits and a decrease of BOLD fluctuation in the main nodes of the DMN using ALFF analysis and correlated them to cognitive dysfunction and changes in sleep parameters (Li et al., 2016b).

## 4 Applications of rs-fMRI in insomnia disorder

Insomnia is the most common sleep disorder, characterized by nocturnal and diurnal symptoms with a principal complaint of dissatisfaction with sleep quality or duration (Morin et al., 2015). Patients commonly complain of difficulties in initiating sleep at bedtime, frequent or prolonged awakenings, or early-morning awakening with an inability to return to sleep (Morin et al., 2015). Diagnosis is usually made when sleep complaints are present for more than three nights per week and last for more than three months (Morin et al., 2015). The bidirectional relationship with depression, anxiety and pain is recognized (Morin et al., 2015). The state of research in insomnia is still in its infancy and it is hampered by the heterogeneity of the disorder, which might reflect different underlying causal mechanisms (Morin et al., 2015). Nonetheless, rs-fMRI studies in insomnia disorder point to several intriguing functional abnormalities, as demonstrated in the studies reported here (Table 1).

### 4.1 Abnormalities of functional networks in insomnia disorder

Using independent component analysis (ICA) and a dual regression approach, Chen et al. (2014) have evaluated findings of simultaneous rs-fMRI and electroencephalography (EEG) (Chen et al., 2014). Their findings pointed out that patients with insomnia compared with controls had increased synchronicity of bilateral anterior insula with SN (Chen et al., 2014). Moreover, FC between the insula and SN was positively associated with self-reported alertness and negative affect (Chen et al., 2014). In addition, the time-course of the BOLD signal in the anterior insula was correlated with EEG gamma frequency power during rest in the patients group. These results highlight a potential role of the SN and insula in insomnia (Chen et al., 2014). The insular cortex integrates emotional and bodily states, and its dysfunctional connectivity with other brain areas may underlie the vigilance, subjective distress, and poor sleep continuity of patients (Chen et al., 2014, Uddin, 2015). Previous studies have suggested that the insula and left medial PFC are critical regions in maintaining sleep (Chuah et al., 2006, Koenigs et al., 2010). In another study, the lower connectivity between the amygdala and insula, striatum and thalamus was reported, suggestive of dysfunctionality of emotional circuits in patients with insomnia (Huang et al., 2012). Moreover, the observed higher FC between the amygdala and premotor and sensorimotor cortex has been suggested as a compensatory mechanism. The authors also found a positive correlation between the Pittsburgh Sleep Quality Index (PSQI), which measures subjective sleep quality, and FC of the amygdala with the premotor cortex in the patients group (Huang et al., 2012). In addition, increased amygdala activity in response to the presentation of sleep-related stimuli has been reported in patients with insomnia, suggesting an important role of the amygdala in insomnia-related emotional disruption (Baglioni et al., 2014).

In a recent study of 42 insomnia patients and 42 healthy controls by Nie et al. (2015), eight regions within the DMN were defined and region-to-region FC analysis was applied (Nie et al., 2015). The patients showed significant decreased FC between the medial PFC and the right MTL, and also between the left MTL and the left inferior parietal cortices (Nie et al., 2015). Of note is that reduced synchronicity between frontal and posterior hubs of the DMN has previously been shown during sleep (Horovitz et al., 2009). It has been argued that DMN might play a critical role in conscious awareness (Horovitz et al., 2009). Moreover, it has been shown that sleep deprivation, such as might occur in insomnia leads to aberrant stability and function of the DMN (De Havas et al., 2012, Gujar et al., 2010). The findings of another study suggested that greater waking connectivity between the retrosplenial cortex and hippocampus and various nodes of the DMN is associated with lower sleep efficiency, lower amounts of REM sleep and greater sleep-onset latency (Regen et al., 2016). Another study explored FC of the superior parietal lobe as an important region in spatial working memory, which is often impaired in insomnia patients (Li et al., 2014). Group comparison demonstrated increased FC between the bilateral SPL and several DMN areas including the right anterior cingulate cortex (ACC), left PCC, right splenium of the corpus callosum, pars triangularis, insular lobe and also decreased FC between the SPL and right superior frontal gyrus in patients (Li et al., 2014).

### 4.2 Regional abnormalities in insomnia disorder

Regional homogeneity (ReHo) analysis has also been successfully applied to assess functional alterations in insomnia disorder (Dai et al., 2015, Dai et al., 2014, Wang et al., 2015). For example, it has been reported that subjects with insomnia had higher ReHo in several brain areas including the left insula, right ACC, bilateral precentral gyrus, left cuneus, and lower ReHo in the right middle cingulate cortex and left fusiform (Wang et al., 2015). In one study, the ReHo scores suggested abnormal spontaneous activities especially in emotion-related areas in insomnia patients (Wang et al., 2015). It has been suggested that this might present the intrinsic functional architecture of insomnia and its clinical features (Fig. 3) (Wang et al., 2015). Similarly, Dai and colleagues performed ReHo and ALFF analyses in two separate studies and highlighted gender differences in patients with chronic insomnia (Dai et al., 2015, Dai et al., 2014). They found increased ReHo in the left fusiform gyrus and decreased ReHo in bilateral cingulate gyrus and right cerebellum anterior lobe. These results were different between genders, i.e. female patients had higher ReHo in the right superior temporal gyrus and lower ReHo in the bilateral medial frontal gyrus, subcallosal gyrus and anterior cingulate. The authors observed a positive correlation between behavioral scores of several sleep-related questionnaires and homogeneity of the fusiform and a negative correlation between the behavioral scores and homogeneity of the frontal regions (Dai et al., 2014). The same authors also explored ALFF alterations in insomnia patients (Dai et al., 2015). They observed increased ALFF in the temporal and occipital lobes in all insomnia patients compared to good sleepers. Furthermore, different ALFF patterns between genders in the bilateral cerebellum, limbic areas, left premotor cortex, and left dorsolateral PFC were shown (Dai et al., 2015). In particular, women with insomnia showed lower ALFF in the cerebellum and frontal lobe. However, men had lower ALFF in the occipital lobe (Dai et al., 2015). Finally, a negative link between the chronicity of insomnia with ALFF in inferior frontal regions, and excessive sleepiness with ALFF in the left inferior parietal lobule was reported, leading the authors to further argue in support of the hyperarousal hypothesis in insomnia disorder (Li et al., 2016a).

## 5 Major intrinsic networks in sleep disorders, similarities and possible differential clinical biomarkers with MDD

**Fig. 4:**
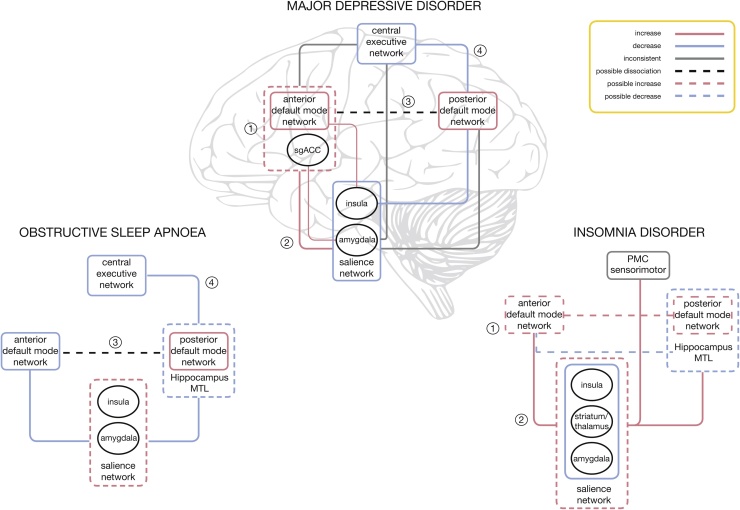
Comparison of the blueprint fingerprint of within- and between-network connectivity changes in sleep disorders, obstructive sleep apnoea and insomnia disorder, with their major neuropsychiatric comorbidity, major depressive disorder (adapted from (Mulders et al. (2015))*.* A within-network increase/decrease in connectivity is presented with pink/blue outlines; lines (pink/blue) between networks represent a between-network increase/decrease in connectivity. Grey lines correspond to inconsistent or unclear findings. Black ellipses represent key nodes related to connectivity. Numbers represent main findings previously reported for the major depressive disorder: (1): increase in the anterior DMN connectivity and inclusion of sgACC within the anterior DMN, (2): increased connectivity between the anterior DMN and SN, (3): changed connectivity between the anterior and posterior DMN, (4): decreased connectivity between the posterior DMN and CEN (Mulders et al., 2015). *Abbreviations*: *CEN: central executive network; DMN: default-mode network; MTL: medial temporal lobe; PMC: premotor cortex; SN: salience network; sgACC: subgenual anterior cingulate cortex.* (For interpretation of the references to color in this figure legend, the reader is referred to the web version of this article.).

Network models have been increasingly recognized as useful tools to study core intrinsic activity features and clinical biomarkers of major neuropsychiatric disorders (Arbabshirani et al., 2013, Dickerson et al., 2004, McCabe and Mishor, 2011, Tahmasian et al., 2016b, Vemuri et al., 2012) (Fig. 4). Most of the findings in OSA and insomnia disorder discussed in previous paragraphs implicate one of three major neural networks: the DMN, the CEN and the SN. The three major intrinsic networks represent the brain’s function during rest, cognition, autonomic and emotional processes, all of which are essential processes that have been reported to be altered in both disorders (Mulders et al., 2015) (Fig. 3, Fig. 4). Perhaps unsurprisingly, the same networks have also been recognized as important in MDD (Mulders et al., 2015), one of the neuropsychiatric disorders most frequently linked with both sleep disorders (Morin et al., 2015, Rosenzweig et al., 2017). So far, studies of insomnia and OSA have suggested several differential fingerprint correlations between symptomatology and the abberant connectivity (see Fig. 3). Broadly speaking, it appears that severity of OSA is closely correlated to changes in the frontoparietal network (CEN) (Zhang et al., 2013). On the other hand, there appears to be a strong corelation between severity of insomnia and changes in the DMN activity (Regen et al., 2016). Furthermore, affective symptoms appear to be more closely connected with aberrations of the SN in patients with insomnia (Chen et al., 2014), and conversely, dysphoric, anixety and hypothymic symptoms of patients with OSA appear to be related to changes in connectivity of the DMN (Zhang et al., 2015). Moreover, both, affective and cognitive symptoms of OSA appear to be strongly linked to functioning of the DMN (Zhang et al., 2015). This is of particular note, given the oft-reported dychotomy between objective findings and subjective symptoms experienced by patients with sleep disorders (Rosenzweig et al., 2017). It has been previously argued that cognitive deficits in patients with depression should be assessed by asking them to integrate piecemeal information into coherent mental representations, as they may not be always observable in measures of more basic memory functions (Brzezicka, 2013). Interestingly, this pattern has been consistently replicated on dysphoric as well as depressed patients (Brzezicka, 2013). The integrative informational ability likely also represents a core mechanism behind reasoning and working memory processes that relie on effective fronto-parietal network interactions (Brzezicka, 2013). The major task of this network is in attentional selection of the relevant stimuli, and any disturbance in this network is likely to have a domino effect on other intrinsic networks, including the overlapping CEN (Mulders et al., 2015, Uddin, 2015). It can therefore be argued that malfunctioning of the CEN likely contributes to executive deficits in some patients with OSA. The listed correlational findings also appear to be in line with data from other structural and EEG studies. Similarly, the imposed state of impaired cognitive control likely gives rise to other frequent and debilitating symptoms of depression, such as persistent rumination (Brzezicka, 2013). On the other hand, as shown in Fig. 3, it seems that severity of insomnia correlates more closely with the abberant connectivity in the DMN circuitry (Regen et al., 2016). Whilst directional causation of this corelation is far from clear, the finding itself seems well supported by other neuroimaging modalities (Tahmasian et al., 2016a). It is likely that hyperactivity of this network, and its inability to deactivate appropriately, may interject to abberant sequential architectural dissociative cascade (Horovitz et al., 2009) and lead to dysfunctional thalamocortical sleep rhythms (Tagliazucchi and Laufs, 2014). This can then contribute to deficits in initiation, maintenance and consolidation of sleep, some of the major clinical complaints in insomnia (Morin et al., 2015).

It has long been argued that the distinction between cognition and emotions is artificial, and that those two phenomena are closely related on the behavioral as well as neural level (Brzezicka, 2013, Davidson, 2004). From the clinical point of view, it is of paramount to elucidate brain networks behind neurocognitive and psychiatric symptoms in sleep disorders, given their clinical value as biomarkers of disease gravity and theraputical validity. It is then perhaps of particular interest to draw parallels and to learn from rs-fMRI studies of MDD (Hamilton et al., 2012, Miller et al., 2015, Mulders et al., 2015), itself the second leading cause of disability worldwide (Mulders et al., 2015). To date the major findings in depression can be summarised by four dominant features: (1) increased connectivity within the anterior DMN, (2) increased connectivity between the SN and the anterior DMN, (3) changed connectivity between the anterior and posterior DMN and (4) decreased connectivity between the posterior DMN and CEN (for a more in-detail review refer to Mulders et al. (2015). These findings also correspond to the current understanding of depression as a network-based disorder (Mulders et al., 2015). Perhaps unsurprisingly, the synthesis of findings in two major sleep disorders demonstrates striking similarity with this fingerprint pattern (see Fig. 4). Although findings differ between studies, the few consistent findings to date would appear to suggest that patients with insomnia share in the first two (Chen et al., 2014, Wang et al., 2015), and the patients with OSA in the latter two (Li et al., 2016b, Park et al., 2016a, Peng et al., 2014, Zhang et al., 2013) dominant features of abberant network activity reported in MDD (Mulders et al., 2015) (Fig. 4). More specifically, the findings of the studies listed in this review could be taken to suggest that clinical symptomatology of insomnia might be overwhelmingly underwritten by abberant hyperconnectivity within the anterior DMN and its connectivity with the SN (Chen et al., 2014). This emphasis on the anterior DMN is noteworthy as whilst both the anterior and posterior parts of the DMN are related to spontaneous or self-generated cognition, the anterior DMN is more related to self-referential processing and emotion-regulation, partly through its strong connections with limbic areas such as the amygdala (Mulders et al., 2015). Hyperactivity of the amygdala, especially in response to sleep related stimuli, has been demonstrated in patients with insomnia (Morin et al., 2015) and could underlie the negative bias experienced by patients in relation to sleep-related stimuli. Similarly, changes in connectivity between the anterior DMN and SN may constitute an increase in top-down modulation of limbic hyperactivity, bottom-up interference of self-processing regions, or both, as has been previously argued to occur in depression (Mulders et al., 2015). Surprisingly, connectivity of the amygdala with striatum, including head of caudate, and other brain regions such as insula and thalamus, appears to be decreased in insomnia (Huang et al., 2012). This could be of clinical importance as it might indicate an inability to control and entrain amygdala responsivity by other limbic regions (Goldstein and Walker, 2014). It may also suggest an increased coupling and inteference by other regions, such as the fight/flight adrenergic brainstem centre of the locus coeruleus (Goldstein and Walker, 2014). Similar abberant connectivity with locus coeruleus has been previously shown to occur in studies of forced sleep restriction (Motomura et al., 2013). Taken together, this pattern of activity might also explain why in insomnia affective symptomatology and hyperarousal are linked with abberations in the SN, and not with the DMN.

In contrast, the findings of OSA studies appear to highlight the role for the decreased synchronicity and connectivity between the anterior and posterior DMN, and between the posterior DMN and hippocampus and the rest of frontoparietal network (Fig. 4). The posterior DMN has been implicated in both consciousness and memory processing through its relation to the hippocampal formation (Mulders et al., 2015), and the abberant functioning of this part of the network is in line with other structural studies of OSA (Tahmasian et al., 2016a). In line with the role of the posterior DMN in awareness and directed attention (Leech and Sharp, 2014), and the role of the CEN in higher cognitive functioning (Corbetta and Shulman, 2002), the change in their interaction could underlie difficulty in switching from an internally directed state in which the DMN is dominant, to an externally directed state in which the CEN is dominant and attention is directed toward outward stimuli (Uddin, 2015). Several authors have suggested that the insular cortex might be crucial for this shift in network-dominance (Uddin, 2015), which is supported by the increased connectivity of the insula with the anterior DMN and decreased connectivity with other networks (Uddin, 2015) (Fig. 3, Fig. 4). In support of this view, in a recent meta-analysis of structural and funtional studies, significant deficits in this region have been highlighted (Tahmasian et al., 2016a). Apart from cognitive deficits that can arise from this abberation, this feature implies problems in sustained and divided attention that arguably then contribute to well recognized driving difficulties in patients with OSA, even without excessive daytime somnolence (Rosenzweig et al., 2017).

### 6 Strengths and limitations of rs-fMRI

It is widely accepted that rs-fMRI represents state of the art in the assessment of intrinsic neuronal activity (Tagliazucchi and Laufs, 2014). During rs-fMRI, in the absence of any externally induced task, distributed brain regions exhibit coherent low-frequency fluctuations binding them together into identifiable functional networks (Fig. 1) (Fox and Raichle, 2007). Similarly, it has been generally accepted that rs-fMRI presents a promising tool in the search for functional biomarkers of a variety of neurodegenerative, neuropsychiatric, pain and sleep disorders (Tagliazucchi and Laufs, 2014, Tahmasian et al., 2015a). However, there are several important limitations of this technique that need to be accounted for. Firstly, resting-state is an uncontrolled, insufficiently understood condition that arguably differs in any one person during various degrees of wakefulness, during different times of the day, and during differential baseline “consciousness” thought processes (Muto et al., 2016, Tagliazucchi and Laufs, 2014). It follows that in sleep disorders, where it is specifically difficult to control for degree of somnolence and ‘micro-sleeps’ even with co-registration with EEG, it might be particularly difficult to account for the exact degree of wakefulness (Tagliazucchi and Laufs, 2014). By a way of example, using self-report questionnaire (Amsterdam Resting-State questionnaire), it has been shown that sleepiness level is associated with FC scores within the DMN, visual, and sensorimotor network (Deco et al., 2014, Stoffers et al., 2015). Also, it has been demonstrated that even in those without known predisposing disorders, 30% of individuals could not stay awake after three minutes and 50% of them had at least one epoch of sleep (Tagliazucchi and Laufs, 2014). Hence, FC alteration due to falling sleep is an important cofounding issue in patients with sleep disorders as they have higher daytime sleepiness (Morin et al., 2015, Rosenzweig et al., 2017) and might fall in sleep easier than healthy subjects in the scanner. Secondly, the majority of neuropsychiatric conditions, including the two major sleep disorders reviewed here, are multifactorial disorders, with predisposing genetic, promoting epigenetic and environmental, and precipitating acute factors (Morin et al., 2015, Rosenzweig et al., 2017). Hence, the issue of directional causation, the classical chicken or egg problem, might be particularly difficult to decipher here. Namely, the chronicity of sleep issues, homeostatic adaptive and maladaptive processes to disrupted sleep and idiosyncratic susceptibility are all contributing to an endogenous neuronal activity fingerprint at any point in time (Rosenzweig et al., 2017).

Despite all these issues, technically, a paradigm-less experimental setting of rs-fMRI appears especially suitable for building multicenter databases whilst minimizing any confounding influence of local clinical settings (Mulders et al., 2015, Tagliazucchi and Laufs, 2014). However, it is crucial to acknowledge all technological limitations, some commonly shared with other functional imaging methods (Tagliazucchi and Laufs, 2014). For example, it has been recognized that the non-neuronal physiological signals such as respiration and cardiovascular pulsatile rhythms may introduce noise and lead to misinterpretations of resting-state BOLD findings (Birn et al., 2006, Liu et al., 2006). Similarly, white matter and cerebrospinal fluid signals are other potential sources of noise, and they should be removed in the preprocessing of images (Fox et al., 2005). In that respect there is an ongoing discussion in the field regarding the importance of the global signal regression approach, which can remove respiratory and pulsatile noise from the resting-state BOLD signal but it can also introduce anti-correlations between regions, and increase the number of false negatives (Murphy et al., 2009).

Another critical unsolved technical issue in rs-fMRI is the issue of artefacts induced by head motion, which can introduce systematic noise in the BOLD signal. Satterthwaite et al. (2013) highlighted the fact that improved preprocessing and motion correction provides better results (Satterthwaite et al., 2013). They suggested that head motion of subjects should be explicitly reported as an important outcome measure in all rs-MRI studies (Satterthwaite et al., 2013). Hence, the onus is on all the future studies to employ careful preprocessing and verifiable statistical analyses as critical steps to avoid misleading results. Moreover, in order to increase both sensitivity and specificity of rs-fMRI studies, the brain state should be determined and accounted for in the related analysis strategies and results should be critically reviewed for false positives originating from unstable vigilance levels, especially if the state of wakefulness remains obscure (Tagliazucchi and Laufs, 2014). Finally, in a recent study, significant circadian rhythmicity of brain responses in all brain regions except in DPFC, including the thalamus, head of the caudate, and putamen, was shown (Muto et al., 2016). Hence, it follows that apart from homeostatic influences, it is also the circadian control over intrinsic activity that should be accounted for in any future studies (Muto et al., 2016).

Moreover, it should also be noted that rs-fMRI is measuring oxygen consumption and therefore is an indirect measurement of neural activity (Tagliazucchi and Laufs, 2014). Thus, the combination of fMRI with other brain mapping modalities (e.g. EEG) may provide more comprehensive information to explore the pathophysiology of sleep disorders (Tagliazucchi and Laufs, 2014). Beyond these applications, rs-fMRI is a potent, non-invasive tool for clinical purposes, e.g. to separate patients from healthy individuals or subtypes of particular sleep disorder or to observe the effects of different medication in functional networks (Mulders et al., 2015, Tagliazucchi and Laufs, 2014).

## 7 Conclusions and future directions

The studies listed above highlight the important role of rs-fMRI as a promising non-invasive method for better understanding of the pathophysiology of the two most prevalent sleep disorders, insomnia disorder and OSA. The underlying core neural mechanisms and neurocircuitry of both disorders are still matter of some debate in the field. It is increasingly recognized that, due to the wide range of idiosyncratic vulnerabilities and multiple epigenetic and genetic adaptive and maladaptive mechanisms (Baril et al., 2017) at play, it is very difficult to predict specific future risk for any individual patient. In addition, an important issue of gender differences, and the potential impact of sex hormones on pathophysiology of both disorders during reproductive age in women, whilst recognized in the field (O’Halloran et al., 2016), is far from being fully explained and documented. Similarly, the diversity and the diffuse nature of reported neurocognitive deficits, make the panacea “cure-all” treatment approach in these disorders less likely. Elucidating the individual resting-state neurocircuitry connectivity and presence of any neural network dysfunction might be the most appropriate first step for any personalized medicine approach in treatment of any sleep and neuropsychiatric disorder. Notwithstanding this, the emerging blueprints of the specific patterns of dysfunctional connectivity for both of these sleep disorders are emerging (see Fig. 4), and they appear to bear striking similarity with specific, if diverse, aspects of aberrant activity in MDD. As such, they may provide a potential clinical biomarker of functional deficits frequently reported by patients, as well as guide future therapeutic efforts. Future studies using standard preprocessing and statistical analysis are warranted in this field (Tagliazucchi and Laufs, 2014). Such studies should focus on the progression and trajectory of disorders and should aim to evaluate potential treatment effects. It has been argued that adopting within-group repeated measures and randomized controlled cross-over designs in future studies of sleep disorders may allow for more precise phenotyping and assessment of clinically useful neural markers that underpin functional daytime impairments (Vakulin and Stevens, 2016). Moreover, the recent advent of hybrid positron emission tomography (PET)/MR scanners may enrich our ability to investigate connectivity by introducing the concept of metabolic connectivity whilst enabling insights on the physiological and molecular bases underlying high-level neural organization (Aiello et al., 2016, Otte et al., 2016, Riedl et al., 2014, Tahmasian et al., 2015b). This multimodal imaging approach might be of particular clinical value in future studies of glymphatic system (Xie et al., 2013). The glympathic system has recently been implicated in the clearance of toxic metabolites in rodents during sleep (Xie et al., 2013), and an indirect clinical evidence could be arguably taken to suggest its contributory role in pathophysiology of the two major sleep disorders, insomnia disorder and OSA (Cedernaes et al., 2017, Ju et al., 2016, Mander et al., 2016). In conclusion, an increasing recognition of the complexity of pathophysiological processes in sleep medicine is driving the move towards more personalized diagnosis and treatment approaches where the rs-fMRI may present as a pivotal future clinical tool.

## Contributors

Authors HK, MV, MT and IR wrote and planned the review. All authors contributed equally to the revision of this manuscript. The authors apologize to all the colleagues whose outstanding work could not be cited due to space limitations.
